# Psychiatric stigma in patients with comorbid hiv infection

**DOI:** 10.1192/j.eurpsy.2021.1356

**Published:** 2021-08-13

**Authors:** M. Khobeysh, N. Lutova

**Affiliations:** The Integrative Pharmaco-psychotherapy Of Mental Disorders, V.M. Bekhterev National medical research center for psychiatry and neurology, Saint-Petersburg, Russian Federation

**Keywords:** stigma of mental illness, Internalized stigma, HIV infection, Schizophrenia spectrum disorders

## Abstract

**Introduction:**

HIV-positive patients with schizophrenia spectrum disorders experience burden of double stigma. Comorbid pathology may alter structure of stigma and shall be considered in development of individual destigmatization programs.

**Objectives:**

Study of psychiatric stigma features in HIV-positive and HIV-negative patients with schizophrenic disorders.

**Methods:**

ISMI (Ritsher et al., 2003), PDD (Link et al., 1991) – to study stigma in 70 patients divided into three groups with respect to their diagnosis (I — F20.x, II — F21.x, III— F2x+HIV); BPRS (Overall & Gorham, 1962) – to assess psychiatric status, RSAS (Eckblad et al., 1982) – to assess anhedonia. Dispersion analysis (Kruskal and Mann–Whitney tests), Spearman and Pearson correlation were used.

**Results:**

Patients with comorbid HIV-infection showed increased level of perceived stigma, although they resisted the stigma internalization better than others did (Table 1).

**Figure:**
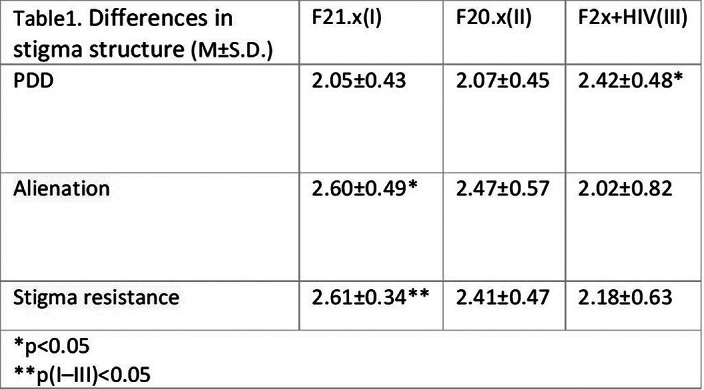

Patients with schizotypal disorders and patients at early stages of HIV infection experienced the most alienation and frailty to internalization of stigma (Tables 1, 2).

**Figure:**
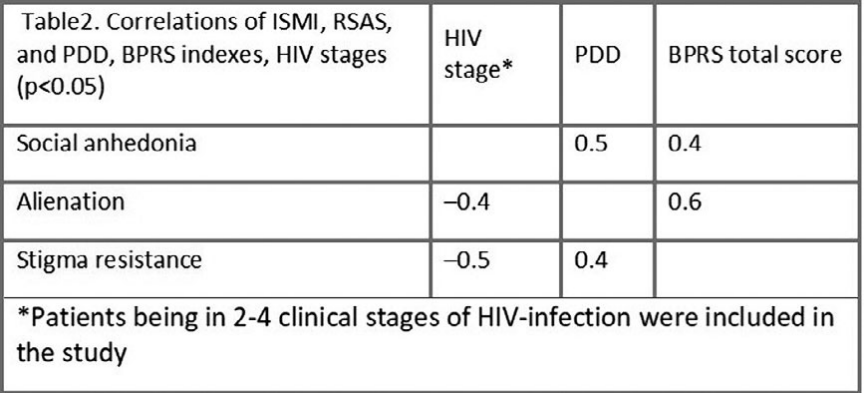

Correlation relationship between social anhedonia and perceived stigma (r=0.5, p<0.05) observed in patients with HIV infection.

**Conclusions:**

Comorbid HIV infection in psychiatric patients contributes to the psychiatric stigma structure. Differentiated approaches in rehabilitation of HIV-positive mental patients should be used.

